# Development and Geometrical Considerations of Unique Conductive and Reversible Carbon-Nanotube Hydrogel without Need for Gelators

**DOI:** 10.3390/gels10070457

**Published:** 2024-07-12

**Authors:** Ryo Ogawa, Ryota Arakaki, Takahide Oya

**Affiliations:** 1 Graduate School of Engineering Science, Yokohama National University, Yokohama 240-8501, Japan; 2 Semiconductor and Quantum Integrated Electronics Research Center, Institute for Multidisciplinary Sciences, Yokohama National University, Yokohama 240-8501, Japan

**Keywords:** carbon nanotube, carbon nanotube dispersion, hydrogel, dispersant, conductive gel

## Abstract

We propose a new type of CNT hydrogel that has unique conductive and reversible characteristics. We found in previous studies that CNT dispersions became gelatinous without any gelators when a specific CNT was combined with a specific dispersant. This hydrogel has conductive properties derived mainly from the CNTs it contains; and even after gelation, it can be returned to a liquid state by ultrasonic irradiation. Furthermore, the liquid is gelable again. In this study, we prepared several types of CNTs and several types of dispersants, experimentally verified the possibility of gelation by combining them, and geometrically investigated the gelation mechanism to determine how this unique hydrogel is formed. As a result, we found that the experimental results and the theory examined in this study were consistent with the combination of materials that actually become hydrogels. We expect that this study will allow us to anticipate whether or not an unknown combination of CNTs and dispersants will also become gelatinous.

## 1. Introduction

In recent years, nanotechnology research has made great progress. Among such research, studies on nanocarbon materials, such as fullerene, carbon nanotube (CNT), and graphene, are being conducted in a wide range of fields—from fabrication techniques to applications. In particular, CNTs, discovered in 1991 [1], are known for their high chemical stability, mechanical toughness, high electrical and thermal conductivity, and metallic and semiconducting electrical properties [2,3,4,5,6,7]. Because of the various beneficial characteristics described above, there are great expectations for the practical application of various objects using CNTs [8]. However, because CNTs are generally very small, with a diameter of only a few nm and a length of only a few μm, and because most commercial products on the market are in powder form or in water dispersion, they are difficult to handle as they are, making it difficult to develop applications for them. One solution to this problem is to mix CNTs with other materials and handle them as “CNT composite materials”. By making a composite material, handling becomes easier, and the functions of CNTs can be used in that form [9,10,11,12].

Recently, soft materials that contain CNTs and can take advantage of the electrical conductivity and other properties of CNTs have been pioneered. A typical example is CNT gel. Generally, gels hold solvent molecules inside a network structure formed by polymers and other materials. The structure is formed by the cross-linking of solute molecules such as polymers [13]. The CNT gel was first discovered by Fukushima et al. in 2003 when they ground CNTs in an ionic liquid and found by chance a gel-like composite [14]. An important point in constructing the gel structure is how the backbone is cross-linked. In the CNT gel, according to [14,15], the ions that compose the imidazolium-ion-based ionic liquid are stacked on the CNT surface and adsorbed on other CNT surfaces to form a cross-linked structure. In concrete, when CNTs and ionic liquid are ground in a mortar, the bundles of CNTs are loosened, and the detached CNTs are coated with imidazolium ions that form the ionic liquid while the imidazolium ions align between the CNTs to form cross-links. The alignment of imidazolium ions is due to intermolecular interactions and is not as strong as covalent bonding. This gel became known as Bucky Gel, and it was later found that it could be used for various applications such as actuators, dielectrics, and electric double-layer capacitors [16,17,18,19]. In subsequent studies, guest-responsive CNT hydrogels [20,21,22,23,24,25,26,27] and thixotropic CNT gels [28,29,30,31] have been reported as CNT-containing gels other than Bucky gels, each with different gelation mechanisms and applicability. These gels have been investigated in detail, including gelation mechanisms and characteristics, by advancing various studies [32]. Furthermore, recently, a new type of CNT gel that utilizes PEDOT:PSS, a conductive polymer, has also been reported [33,34]. The common feature of almost all of these gels is that the cross-linking for gelation is derived from the chemical coupling or physical linkage between molecules, including polymers.

As CNT composite materials, we are developing “CNT composite paper [35]” and “CNT composite thread/fabric [36]”, which can be easily handled as “familiar objects” with the various features of CNTs. These composite materials have attracted attention as unique and new materials because they have the same processability and deformability as paper, thread, and textile while maintaining the various functions of CNTs. Various applications of the composites are already under investigation, including the feasibility of “paper dye-sensitized solar cells [37]”, “thermoelectric power generating papers/threads [38,39]”, and “paper actuator [40]”. How to prepare CNT dispersions is important in the fabrication of our CNT composite materials. Basically, to obtain a CNT dispersion, a dispersant is needed in addition to CNTs and water [41,42,43,44,45]. This is because CNTs themselves are hydrophobic and cannot be dispersed in water as is. However, most dispersants are insulating, and since they are in between each CNT in a dispersion, they increase the resistance of the sample so that they are a hindrance, for example, to electrical applications. Therefore, we focused on the conductivity of the dispersant itself and found that we could use phthalocyanine-based molecules as a dispersant that had both conductivity and the ability to disperse CNTs in water. We confirmed that by using this to prepare CNT dispersions and using the dispersions to fabricate our CNT composites, the fabricated samples could achieve high conductivity [39]. During the development of this dispersion, we discovered by chance that a combination of specific CNTs and specific dispersants causes the CNT dispersion to the hydrogel. Surprisingly, our CNT hydrogel does not require the polymers and gelators that are usually needed. In our recent studies, it was suggested that the CNT hydrogel is different from other gels in which the polymer constitutes the backbone or cross-linked structure and that the CNTs themselves constitute the backbone, and the hydrophobic interactions between CNTs in water (dispersion) cause the formation of the cross-linked structure [46]. In this paper, we report our investigation and discussion with experimental and geometrical considerations on the gelation of the CNT dispersion and the characteristics of the hydrogel.

## 2. Results and Discussion

As mentioned above, we found in previous studies that CNT dispersions became gelatinous when a specific CNT was combined with a specific dispersant [46]. The CNTs we used at that time were (6,5)-chirality CNTs (SG65i, CHASM, Boston, MA, USA), and the dispersant was a phthalocyanine derivative called C.I. Reactive Blue 21 (Santa Cruz Biotechnology, Inc., Dallas, TX, USA). In this section, we will discuss the gelation of a CNT dispersion, a comparative study of the types of CNTs and dispersants, and an evaluation and discussion of the gelated CNT dispersion.

### 2.1. Experiments on CNT Hydrogelation Consisting Only of CNTs, Dispersants, and Water from CNT Dispersion

Gelation experiments were performed using the combination of CNTs and dispersants described in the following Section 4.1 and the method introduced in Section 4.2. Table 1 shows the results of gelation for each CNT and dispersant combination.

**Figure 1:**
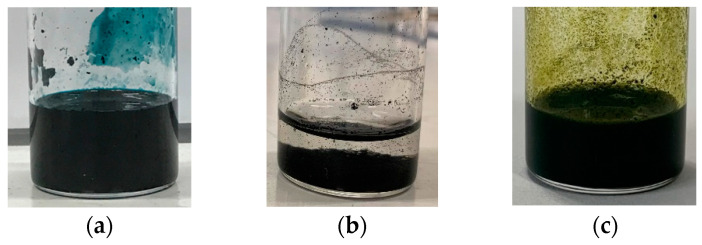
Experimental results of CNT dispersion preparation using (**a**) C.I. Reactive Blue 21, (**b**) 5,10,15,20-Tetrakis(4-aminophenyl)porphyrin, and (**c**) 5,10,15,20-Tetrakis(4-carboxymethyloxyphenyl) porphyrin. (When Cyanocobalamin or SDS was used, same result as in (**a**)).

**Figure 2:**
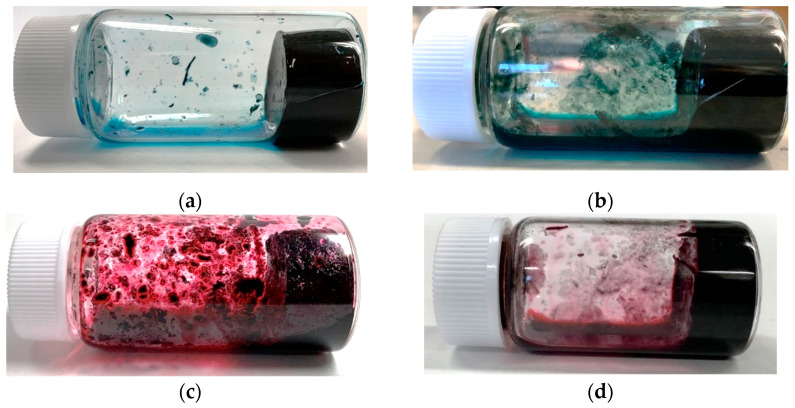
CNT hydrogels produced from a combination of the following: (**a**) (6,5)-chirality CNT and C.I. Reactive Blue 21; (**b**) CG300 and C.I. Reactive Blue 21; (**c**) (6,5)-chirality CNT and Cyanocobalamin; (**d**) CG300 and Cyanocobalamin.

Here, the gelation experiment was conducted using 15 mL of pure water, 25.5 mg (0.17 wt%) of CNTs, and 102 mg (0.67 wt%) of dispersant, based on previous studies [46]. The amount of dispersant used was first checked to ensure that it was sufficient enough to make the CNT dispersion since the preparation of the CNT dispersion was a prerequisite for the experiment. In this trial, as shown in Figure 1, C.I. Reactive Blue 21 (Figure 1a), 5,10,15,20-Tetrakis(4-carboxymethyloxyphenyl)porphyrin (Figure 1c), Cyanocobalamin, and SDS were able to disperse the CNTs in water (at the end of step 2 described in Section 4.2), but 5,10,15,20-Tetrakis(4-aminophenyl)porphyrin failed to disperse the CNTs (Figure 1b). Therefore, we proceeded with the gelation experiment using combinations excluding 5,10,15,20-Tetrakis(4-aminophenyl)porphyrin. The results showed that the CNT dispersion gelatinized if it was consisted of a combination of (6,5)-chirality CNT or CG300 and C.I. Reactive Blue 21 or Cyanocobalamin, as shown in Figure 2. The amount of C.I. Reactive Blue 21 used in this case was 102 mg, and the amount of Cyanocobalamin used was 720 mg (4.57 wt%). Here, we consider that the difference in the amount used is due to the difference in the molecular weight and dispersing ability of each dispersant. We have confirmed more than 50 times by experiment that the dispersion was gelated under those conditions we found as well, and we believe that the gelation conditions are reasonable. These hydrogels have also been found to revert from gel to a liquid state upon ultrasonic irradiation, similar to the results of our previous study [46]. The reason why the hydrogel reverts to a dispersion upon ultrasonic irradiation is that the backbone of the gel is CNTs, and the cross-linked structure is formed by hydrophobic interaction (physical adsorption) between CNTs, as described in Introduction and in detail in Section 2.5 below. Therefore, by applying strong vibrations such as ultrasonic irradiation, this structure is untangled and returns to CNT dispersion, just as when CNTs are made into the dispersion. We also confirmed that this returned dispersion could gelate again by following the same process described in Section 4.2, as we have reported in our previous studies [46]. The power of ultrasonic irradiation during the preparation of CNT dispersion and when returning the hydrogel to the dispersion is exactly the same and of a magnitude that does not destroy the CNTs.

### 2.2. Investigating Response of CNT Hydrogels to Heating Time of CNT Dispersion

As described in Section 4.3, our CNT hydrogels are obtained by heating a CNT dispersion. The results of our investigation of the influence of different heating times on the solidity of the hydrogels are described below. Here, we proceeded with our investigation by focusing on hydrogels made from the combination of (6,5)-chirality CNT and C.I. Reactive Blue 21, assuming that the formation mechanism of the four types of our CNT hydrogels obtained in the previous section is identical (the possible formation mechanism is discussed in Section 2.5).

**Figure 3:**
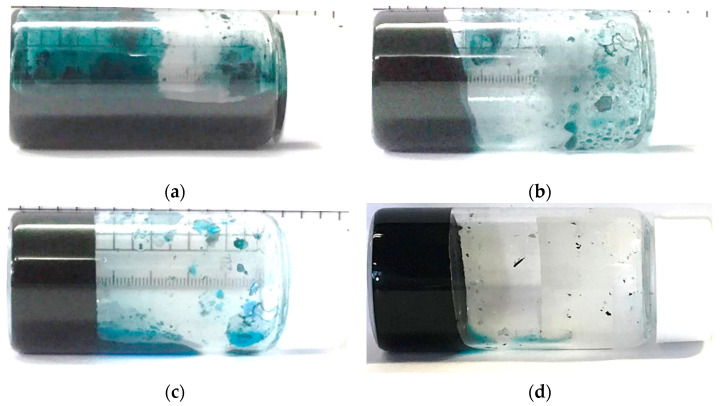
Change in state of our CNT hydrogels with heating time: (**a**) Original CNT dispersion before heating; (**b**) Dispersion after heating at 60 °C for 20 min (gelation); (**c**) Dispersion after heating at 60 °C for 60 min (gelation); (**d**) Dispersion after cooling (**c**) at 0 °C for 180 min (keeping gelation).

Figure 3a–c show the difference in the gelation of the CNT dispersion when the heating time was varied. The dispersion, which was in a liquid state without gelation when no heating was applied (Figure 3a), showed gradual gelation when heating was applied at 60 °C for 20 (Figure 3b) and 60 min (Figure 3c). Table 2 shows an example value of the compressive breaking stress measured for each sample. The dispersion without heating was not measured because it was in a completely liquid state. The longer heating time tends to increase the value of compressive fracture stress, i.e., the CNT hydrogel becomes harder, indicating that the CNT hydrogel gelated by heating. On the other hand, Figure 3d shows a sample that was gelatinized once and cooled at 0 °C for 180 min, which showed no significant change compared with Figure 3c before cooling, and there was no change in stiffness. From the above, it can be said that our CNT hydrogel is a thermosetting gel that gels due to heat but is not thermally reversible. As explained in Section 2.1, the CNT dispersion will be gelated if the experiments are performed under the discovered gelation conditions. In contrast, as discussed in detail in Section 2.5, we have confirmed that our hydrogels show a certain variation in properties for each fabrication. This may depend on the conditions during the preparation of the CNT dispersion, for example. Although the amount of each material can be controlled for preparation of the CNT dispersion, it is hard at this time to precisely control the dispersing condition of CNTs in the dispersion. Even if the dispersion is prepared under the same conditions, the dispersion may contain many CNTs in an isolated state, or it may contain many CNTs in a bundled state. We, thus, consider that the proposed hydrogel will not only have isolated CNTs for the network but also a few bundles of CNTs. Consequently, when multiple samples are prepared and compared, the gel network condition will not necessarily be the same among samples as discussed in the following Section 2.5. It is difficult at this time to simply compare and evaluate the same fabrication conditions. However, there is a common trend in which stiffness changes with heating time, as shown in Table 2. For a strict evaluation of the compressive breaking stress, we consider it necessary to limit the CNT network conditions described above. For example, if we can prepare several CNT dispersions in which the CNTs are completely isolated in the liquid, and if they are transformed into hydrogels as well, then a strict evaluation of the compressive breaking stress will be possible. As the next step of this study, the CNT network condition will be studied, and we will conduct a detailed evaluation of the compressive breaking stresses in the near future. As a simplified evaluation in this study, we believe that our CNT hydrogels definitely tend to depend on the heating condition during gelation for their stiffness.

We also conducted a series of experiments in which the temperature conditions were changed by 10 °C. The results showed that the gelation progressed somewhat faster at 70 °C and 80 °C. However, it was similar to that in the temperature condition at 60 °C. At 90 °C or higher, the water in the dispersion began to evaporate, and the experimental conditions could no longer be maintained, so the evaluation was discontinued. On the other hand, below 50 °C, there were cases of gelation and cases of no gelation, as discussed in Section 2.3 and Section 2.5, which may have depended on the degree of desorption of the dispersant, the conditions of CNTs, including how bundled they are, and (local) concentration changes in the dispersion solution.

### 2.3. Investigating Influence of Dispersion Concentration

As described in Section 4.4, we investigated the gelability of CNT dispersions in terms of the concentration of the dispersions. Table 3 shows the results.

As can be seen from Table 3, the threshold CNT concentration required for gelation is 0.13 wt% (e.g., [CNT]: [C.I. Reactive Blue 21]: [pure water] = 25.5 mg: 102 mg: 20 mL), which is relatively low among other reported CNT gels. For example, typical CNT gels, i.e., bucky gels and thixotropic CNT gels, are around 1 wt%, and guest-responsive CNT hydrogels have been reported to gel at a CNT concentration of 0.2 wt% [14,20,28].

Next, gelation experiments with different concentration ratios of (6,5)-chirality CNTs and C.I. Reactive Blue 21, i.e., [C.I. Reactive Blue 21]/[(6,5)-chirality CNT] = 1, 3, 4.5, 6, 9, and 12 in addition to 4 as discussed above, were conducted. Here, the concentration of CNTs was fixed at 0.1 wt%. Table 4 shows the results. Since C.I. Reactive Blue 21 was used as a dispersant in this study, it was impossible to make dispersions when the concentration was less than three times that of the CNTs due to a lack of the dispersant. When the concentration was more than three times the CNT concentration, it was confirmed that gelation occurred at any concentration. On the other hand, the compressive breaking stress of the samples with 4.5 times and 12 times C.I. Reactive Blue 21 added to the CNTs was measured as an example and was 2.8 kPa and 0.88 kPa, respectively. In other words, the hydrogels tended to become softer as the amount of C.I. Reactive Blue 21 was increased. The sample with 3 times the amount of C.I. Reactive Blue 21 added to the CNTs showed gelation even at room temperature. From the above results, it can be considered that an appropriate amount of dispersant is necessary for the preparation of CNT hydrogels; however, the use of excessive amounts of dispersant inhibits gelation, as indicated by the results showing a tendency for the compressive breaking stress to decrease.

### 2.4. Investigating Electrical Properties of CNT Hydrogels

**Figure 4:**
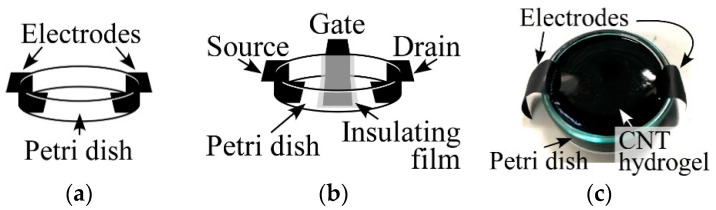
Schematic of prepared petri dishes for measuring (**a**) resistance and (**b**) FET property. (**c**) Fabricated CNT hydrogel in petri dish shown in (**a**). Diameter of petri dish is 4 cm.

As described in Section 4.5, we investigated the electrical properties of CNT hydrogels. For this, we prepared a CNT dispersion using (6,5)-chirality CNTs and C.I. Reactive Blue 21 by following procedure steps 1 and 2 described in Section 4.2. After that, we poured the dispersion into a petri dish with a pair of electrodes at the bottom, as shown in Figure 4a, and heated the whole petri dish following procedure step 3 to create a CNT hydrogel in the petri dish for easy evaluation. Also, to evaluate the field effect transistor (FET) properties of the CNT hydrogel, we poured the dispersion into a petri dish with three electrodes at the bottom, as shown in Figure 4b, to create CNT hydrogels in the dish. Figure 4c shows, as an example, CNT hydrogels actually prepared in the petri dish shown in Figure 4a. In these measurements, a petri dish with a diameter of 4 cm was used, and carbon-based electrodes (conductive tape) were used to prevent chemical reactions with the hydrogel. The width of each electrode was 1 cm. The distance between the two electrodes was 1 cm. In addition, to evaluate the FET properties, an insulating film was inserted between the gate electrode and the hydrogel. For the gate electrode, copper tape was used because it was separated from the hydrogel by an insulating film, so no chemical reaction was expected to occur.

**Figure 5:**
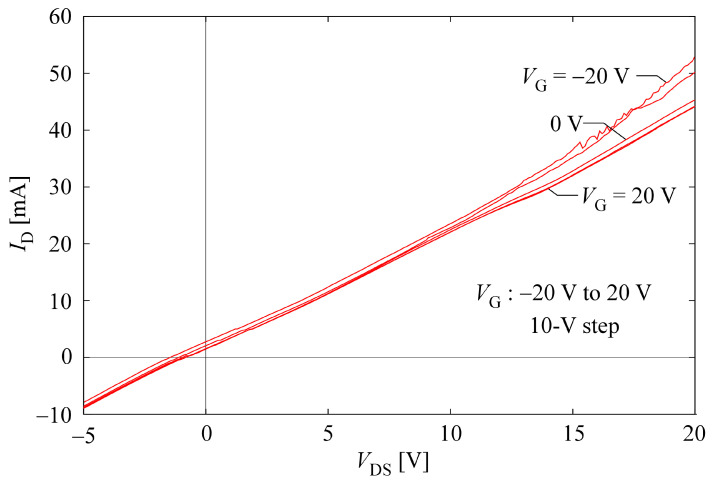
*I*_d_–*V*_d_ characteristic of “gel FET”.

As a result of measurement, we found that our CNT hydrogel had a resistivity of about 8.9 Ω·m (averaged value of five samples), i.e., our hydrogel was a conductive-type gel. Generally, conductivity percolation is evaluated in conductive gels containing conductive materials such as CNTs. In other studies for conductive gels, it has been reported that a clear percolation threshold can be observed [47,48,49,50,51,52]. However, it is difficult to find a clear threshold because our hydrogels obtain conductivity as soon as they are gelated. This is because—as mentioned above—the quantitative balance between CNT and dispersant concentrations, and water is very important for the gelation of the CNT dispersion. If the amount of CNTs is reduced to find the percolation threshold, gelation will not occur. As discussed in the next subsection, the backbone of our hydrogel is considered to be a network of CNTs, so it may be reasonable to consider the conditions for gelation to be the threshold. Since the CNTs used in this experiment have semiconducting properties, we then tried to measure the FET property of our CNT hydrogel by using the method described above. Figure 5 shows the result. Linear changes were strongly caused by the conductive molecule C.I. Reactive Blue 21 and metallic CNTs mixed in as impurities. However, it can also be seen that the magnitude of the current changed slightly in response to the gate voltage being controlled at a high *V*_d_. This trend was also confirmed for the combination of CG300 and C.I. Reactive Blue 21, which showed gelation, and also for hydrogels made with Cyanocobalamin instead of C.I. Reactive Blue 21. Therefore, although the properties are still weak, we consider that we have confirmed the feasibility of “gel FETs”. If we can increase the purity of the semiconducting CNTs and prepare a dispersant that can contribute to gelation without affecting the electrical properties of the CNTs, we believe we can complete the gel FET in the near future.

### 2.5. Discussion with Geometrical Considerations of Changing CNT Dispersion into Hydrogel

As described in Section 2.1, we found that the combination of certain CNTs and dispersants caused the CNT dispersion to the hydrogel. Regarding the combination of CNTs and dispersants used in the dispersions that gelated and those that did not, the following can be considered.

The conditions under which the gelation occurred in this study revealed two common points about CNTs and dispersants. The first was suggested in previous studies [46] but is highly dependent on the diameter of the CNT and the size (core size) of the dispersant. Especially since it was confirmed that combining a CNT with a diameter of about 0.8 nm and a dispersant with a size of 1.5 nm would be gelable, these two sizes are considered to be very significant in this study. According to the supplier’s product specifications, HiPco CNTs with a diameter of 0.8 nm are also contained in the product, but this is considered to be a small amount, so the use of HiPco CNTs would not have resulted in gelation. From this, we found that this CNT hydrogel preparation could be used for CNT diameter separation. Second, the shape of the dispersant is also considered important. The dispersants that contributed to the gelation of the CNT dispersion in this study were C.I. Reactive Blue 21 and Cyanocobalamin, which are substances that have a structure of circular molecules adsorbed on the CNT surface through π–π interactions and have hydrophilic groups at the ends. In particular, they have the characteristic of having a single long chain of molecules. On the other hand, dispersants with a point-symmetric shape, such as 5,10,15,20-Tetrakis(4-aminophenyl)porphyrin, did not cause gelation.

**Figure 6:**
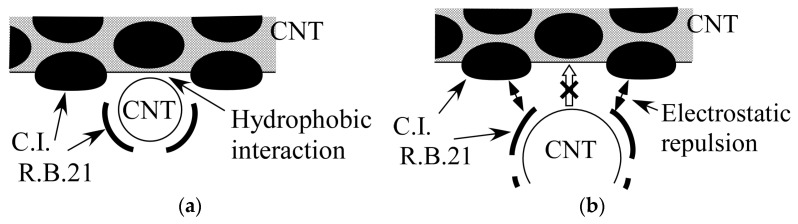
Mechanisms considered for gelation depending on the size difference between C.I. Reactive Blue 21 (C.I.R.B.21) and CNTs: (**a**) Their size is almost the same; (**b**) Not the same.

**Figure 7:**
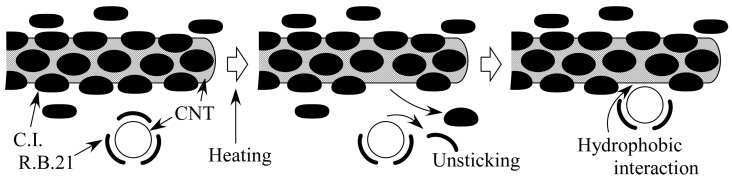
Expected CNT hydrogel gelation mechanism.

From the results obtained in this study, the following mechanism of gelation can be considered. Here, as an example, we discuss the gelation mechanism of the CNT dispersion for the combination of (6,5)-chirality CNTs and C.I. Reactive Blue 21. First, as described in Section 4.1, the size of C.I. Reactive Blue 21 used as the dispersant is about 1.5 nm. The diameter of (6,5)-chirality CNTs is about 0.78 nm; however, the actual diameter is larger because they are covered with the dispersant. C.I. Reactive Blue 21 is adsorbed on the (6,5)-chirality CNT surface through π–π interactions, and the distance between C.I. Reactive Blue 21 and (6,5)-chirality CNTs is estimated to be about 0.33–0.35 nm, which is considered to be the same as the interlayer distance of graphite [53,54]. Therefore, the diameter of (6,5)-chirality CNTs coated with the dispersant is about 1.44–1.48 nm, which is almost consistent with the size of C.I. Reactive Blue 21, which is 1.5 nm. As shown in Table 2, the gelation of the CNT hydrogel is a reaction that proceeds by heating, so we also consider the changes that occur in the dispersion due to heating as follows. It is known that dispersants are released from the surface of CNTs by heating, and CNT dispersions using pyrene derivatives, which adsorb on the surface of CNTs through π–π interactions like C.I. Reactive Blue 21, showed aggregation of CNTs at around 50 °C [55]. This suggests that the CNTs in the dispersion heated to 60 °C in this experiment existed in a state where the dispersant was partially released. In this case, the hydrophobic CNTs exposed after the dispersant is released aggregated with other exposed CNTs due to hydrophobic interaction, but if the sizes of C.I. Reactive Blue 21 and the apparent diameter of the CNTs with C.I. Reactive Blue 21 match as shown in Figure 6a, the exposed hydrophobic portions fit together to form a physical cross-linked structure. On the other hand, when the sizes do not match, as shown in Figure 6b, the electrostatic repulsion of the dispersant prevents the hydrophobic portions from approaching each other and forming a cross-linked structure. For the above reasons, it is considered that heating the dispersion containing CNTs whose apparent size matched that of C.I. Reactive Blue 21 caused gelation, while other CNTs whose size did not match did not. The unique features of CNT hydrogels we found, such as size dependence, do not match the characteristics of the well-known types of gels introduced in the Introduction. For example, if the polymer makes the backbone or cross-linked structure of the gel, the size dependence on CNTs would not be so strong. In contrast, the experimental results described in Section 2.1, Section 2.2, Section 2.3 and Section 2.4 seem to support that CNTs are the backbone in our hydrogels and that the cross-linked parts are composed of CNT linkages due to hydrophobic interactions between CNTs, as mentioned in the Introduction. Figure 7 shows the gelation mechanism of the CNT hydrogel, which can be expected from the above results. After dispersion in water, the CNTs are covered with negatively charged C.I. Reactive Blue 21 and repel each other due to electrostatic repulsion. When the dispersion is heated to 60 °C, some of the C.I. Reactive Blue 21 is detached from the CNT surface by thermal motion, exposing the hydrophobic CNTs. The hydrophobic CNTs aggregate with each other in water due to hydrophobic interaction, and a cross-linked structure is formed by hydrophobic and π–π interactions. This cross-linked structure becomes a three-dimensional network, and as a result, it contains a large number of microscopic spaces. The CNTs that are the backbone of this network are covered with C.I. Reactive Blue 21, which makes their surface hydrophilic. Therefore, it is thought that water is captured in the microscopic spaces created by the network, resulting in a CNT hydrogel. At this time, if an excessive amount of C.I. Reactive Blue 21 is added to the dispersion, the exposed CNTs are immediately covered by C.I. Reactive Blue 21, and as a result, gelation is inhibited. On the other hand, if the amount of C.I. Reactive Blue 21 is small, the possibility of CNTs being covered by C.I. Reactive Blue 21, once exposed, is small, so gelation will occur even at room temperature.

**Figure 8:**
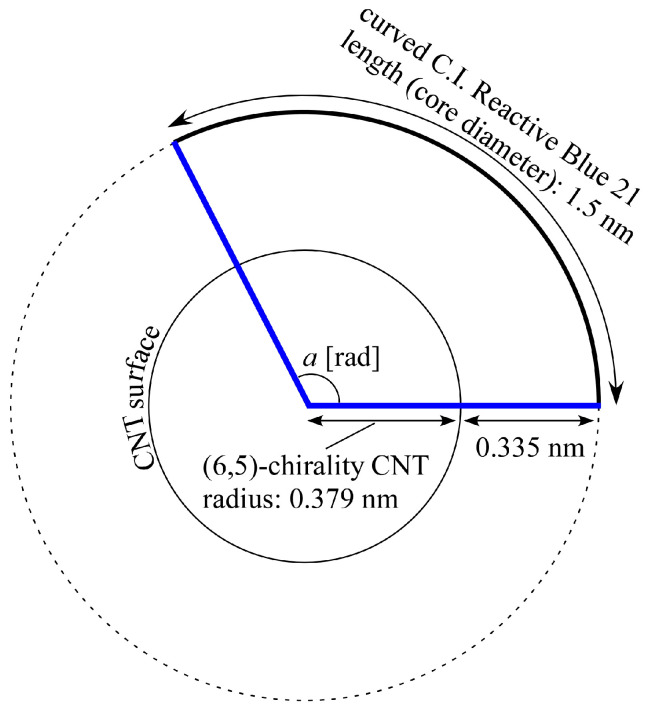
Imaginary view of positional relationship between dispersant (curved sheet) and CNTs from cross-sectional direction of CNT.

Next, a geometrical discussion of the above cross-linked structure is given below. First, it is known that the diameter *d*_t_ [nm] of CNTs can theoretically be expressed as in Equation (1),

(1) $$d_{t}=\frac{\sqrt{3}}{\pi }0.144\sqrt{n^{2}+nm+m^{2}},$$

where *n* and *m* are determined on the basis of the chirality (*n*, *m*) of the CNT [56]. For example, for a (6,5)-chirality CNT, *d_t_* is derived to be about 0.76 nm. This value is generally consistent with that listed in the following Table 5. Next, let us consider the situation where a circular sheet of material, such as C.I. Reactive Blue 21, is adsorbed on the surface of a (6,5)-chirality CNT. For simplicity, we here assume that the sheet-like material is fixed to the CNT surface by π–π interactions and that the distance from the CNT surface to its sheet is about 0.335 nm, referring to the interlayer distance of graphite. Considering that this circular sheet of material is fixed to the CNT surface with a curvature that follows the CNT surface shape, we can estimate how it will cover the CNTs, as shown in Figure 8. From the formula for an arc in geometry, the relationship between the curve length *l* (arc length) of the sheet-like material and the central angle *a* [rad] can be expressed as Equation (2).

(2) a=l(dt2+0.335)

**Figure 9:**
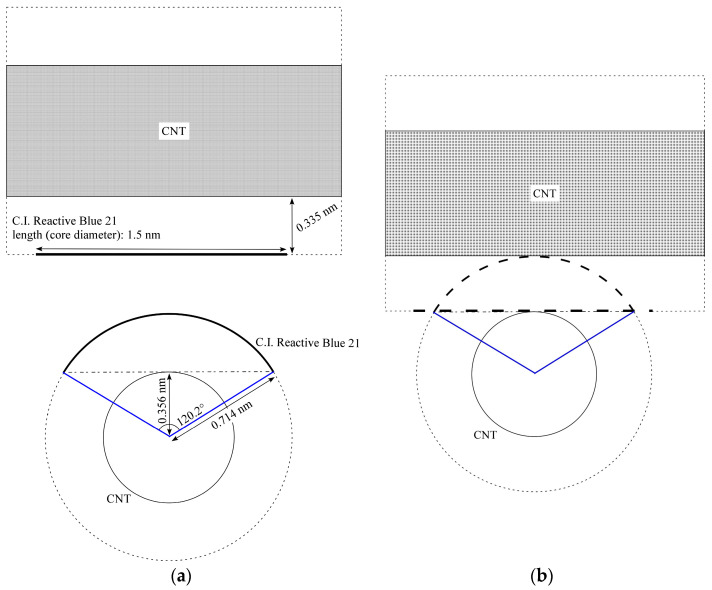
Imaginary geometry of the following: (**a**) Two CNTs with dispersant attached; (**b**) Two CNTs connected after attached dispersants were partly peeled off.

By substituting the (6,5)-chirality CNT diameter and the core diameter of C.I. Reactive Blue 21 (1.5 nm) into this equation, *a* = 2.10 rad = 120.2 deg is derived. This value means that three C.I. Reactive Blue 21 molecules would cleanly cover the entire circumference of (6,5)-chirality CNTs. One of the reasons that CNTs of other diameters did not gel, as shown in Table 1, may be that C.I. Reactive Blue 21 could not cover the CNTs in this clean manner. Next, we assume that only one C.I. Reactive Blue 21 molecule has detached from the CNT surface since the central angle was estimated to be 120.2 deg, and we discuss the geometry related to that area as shown in Figure 9a. Now, let us consider an isosceles triangle with a vertex angle of 120.2 deg and two legs of length 0.714 nm (*d_t_*/2 + 0.335 nm). The height of this isosceles triangle is then calculated to be 0.356 nm. In other words, when one C.I. Reactive Blue 21 molecule detaches from the CNT surface, the center position (in the depth direction) of the exposed CNT is approximately the same as the CNT radius. If another CNT with a detached C.I. Reactive Blue 21 molecule were to exist nearby, and if the CNTs were considered to be linked together as they normally aggregate in water, the distance between the CNTs would be roughly the same as the interlayer distance of graphite. Therefore, it can be said that for CNTs, it would be a spot where they can be linked at the optimum interlayer distance, similar to a wooden structure for building a log cabin, as shown in Figure 9b.

In this study, we have discussed the gelation mechanism of our CNT hydrogels based on a geometrical approach. For a more detailed discussion, a molecular simulation, for example, should be performed. However, we consider that it is difficult to make a detailed evaluation at the present time, including the conducting of molecular simulations, because the number of conditions that need to be clarified is so wide-ranging. That is, although CNTs were considered to be the backbone of the hydrogel in this paper, we considered that CNTs do not necessarily need to be isolated for gelation, and bundles of two or three CNTs are considered to be acceptable. Also, the length of CNTs used originally contains a large variation. In a more extreme case, even if there are more than 5 CNTs bundled together at a point excluding the cross-linked part, gelation may still occur if the part where the cross-links are constructed matches the discussed conditions. As the next step of this study, we will investigate more detailed conditions. Once the detailed conditions of gelation have been clarified to the extent that the molecular simulation can be performed, the mechanism will be discussed in-depth by using the simulation.

## 3. Conclusions

In this study, we reported a more detailed investigation of gelation conditions and an experimental and geometrical consideration of the gelation mechanism in consideration of our previously reported CNT hydrogel studies. From experiments under various conditions, it was confirmed that there is a very strong size dependence for the combination of CNTs and the dispersants that can be gelated. This is supported by theoretical considerations based on geometrical theory and is also expected to provide guidelines for unknown combinations of CNTs and the dispersants that can be gelated. It was also confirmed that the stiffness of the hydrogel varies with the temperature conditions. In addition, unique application possibilities, such as gel FETs, were found. We believe that such unique CNT hydrogels will be utilized in various fields in the near future.

## 4. Materials and Methods

### 4.1. Candidates of CNTs and Dispersants

To investigate the gelation of CNT dispersions, we prepared and used the following CNTs. Table 5 shows the chosen CNTs and their information. Here, we mainly chose CNTs with similar diameters. A multi-walled CNT was also included for comparison.

**Figure 10:**
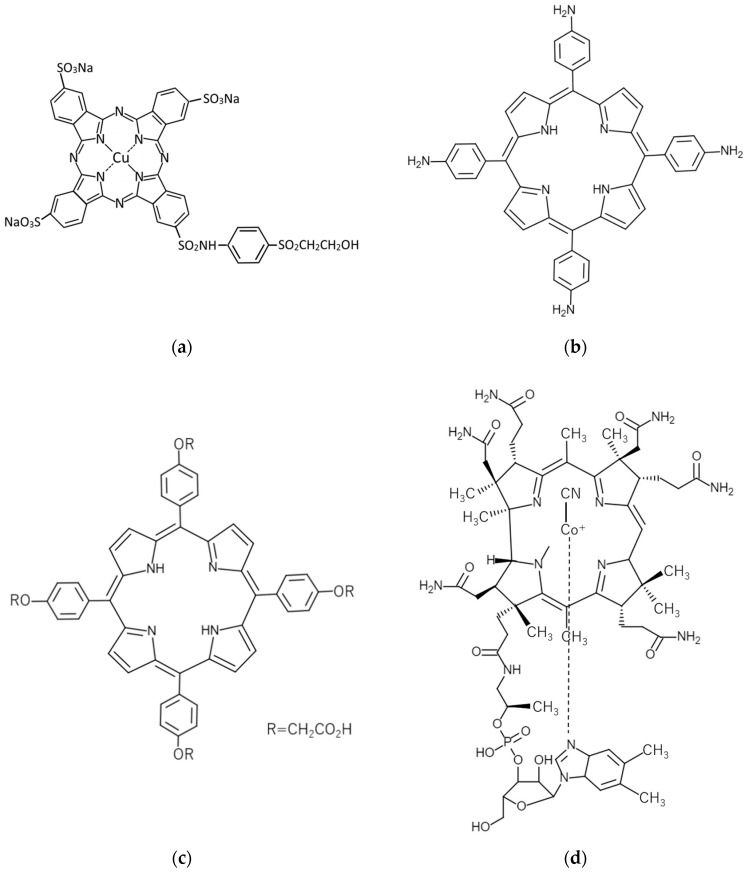
Chemical structural formulas of chosen dispersants: (**a**) C.I. Reactive Blue 21; (**b**) 5,10,15,20-Tetrakis(4-aminophenyl)porphyrin; (**c**) 5,10,15,20-Tetrakis(4-carboxymethyloxyphenyl)porphyrin; (**d**) Cyanocobalamin.

In addition to choosing CNTs as described above, we also prepared and used the following dispersants. Figure 10 and Table 6 show the chosen dispersants and their information. Sodium Dodecyl Sulfate (SDS) was also included for comparison.

**Figure 11:**
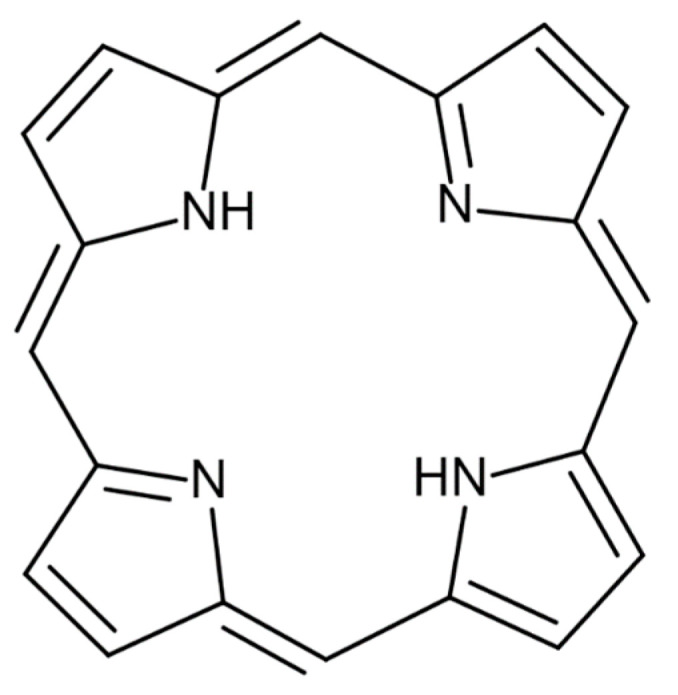
Chemical structural formulas of porphyrin molecule.

C.I. Reactive Blue 21 is a phthalocyanine derivative with a large polycyclic group. It is expected that these polycyclic groups attach to the surface of CNTs through π–π interactions and function as a dispersant. Porphyrin is a substance with a polycyclic aromatic group similar to phthalocyanine. The chemical structural formula of porphyrin is shown in Figure 11. As shown in the figure, porphyrin has a large polycyclic group like phthalocyanine, so it is expected to play a role as a dispersant and to have a molecular size large enough for CNTs to exist and satisfy the above equation. Cyanocobalamin (Vitamin B_12_) was also selected as a candidate because of its similarity to the molecular structure of C.I. Reactive Blue 21. Cyanocobalamin has a polycyclic group and hydrophilic group similar to porphyrin and is thought to attach to the surface of CNTs. Therefore, it is expected to act as a dispersant. Also, like C.I. Reactive Blue 21, it has a structure in which a single carbon chain is connected to a polycyclic aromatic group, so it is considered to have more structural similarities than the porphyrin molecule described above.

### 4.2. Investigating Possibility of Gelation by Combination of CNT and Dispersant Candidates

We verified the gelability of the combinations of the candidate CNTs and candidate dispersants listed in the previous subsection. In this experiment, the following procedure was used for verification. (Basically, the procedure is the same as that for preparing the CNT aqueous dispersion.)

1. A CNT selected from Table 5 and a dispersant selected from Table 6 are stirred into pure water.
2. CNT dispersion is prepared by dispersing the solution prepared in step 1 for 1 h using an ultrasonic homogenizer (UX-050, Mitsui Electric Co., Ltd., Noda, Japan) while cooling the solution to 0 °C.
3. After step 2, the dispersion is heated to 60 °C for 1 h.
4. After step 3, the dispersion is checked to see whether it has gelatinized.

### 4.3. Investigating Response of CNT Hydrogels to Heating Time

**Figure 12:**
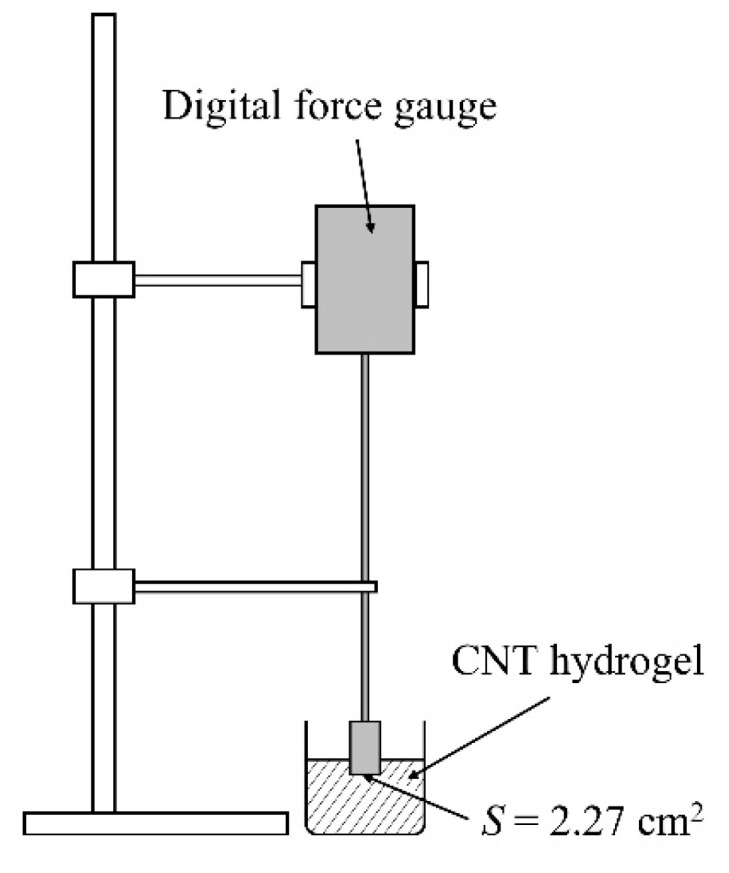
Schematic of apparatus for measuring compressive breaking strength with tip having cross-sectional area of 2.27 cm^2^.

Our CNT hydrogels are thermoplastic gels. In the previous subsection, we described gelation experiments in which the CNT dispersion was heated at 60 °C for 1 h. In this subsection, we describe gelation experiments at other heating times. Here, we prepared three CNT dispersions containing 0.17 wt% of (6,5)-chirality CNT and 0.87 wt% of C.I. Reactive Blue 21. The heating time was differentiated at 0 min, 20 min, and 1 h, respectively, and the change in gel state was confirmed by a simple measurement of compressive breaking stress to confirm the trend. The compressive breaking stress was measured by using the digital force gauge (AD-4932A-50N, A&D Company, Ltd., Tokyo, Japan) shown in Figure 12. When the tip, which has a cross-sectional area of 2.27 cm^2^, is sunk, the compressive force measured by the force gauge increases, and the compressive force is confirmed to yield when the gel is broken. The compressive breaking stress is obtained by dividing the compressive force just before yielding by the cross-sectional area.

### 4.4. Investigating Effect of Dispersion Concentration on Gelation

Here, we focus on (6,5)-chirality CNTs and C.I. Reactive Blue 21 to investigate the gelability of CNT dispersions in terms of the concentration of the dispersions. For this purpose, the ratio of CNT to C.I. Reactive Blue 21 mass was fixed at 1:4, and the gelation was verified by changing the CNT concentration in the dispersion, as shown in Table 7. The method of gelation is the same as described in Section 4.2.

In addition to the above, gelation experiments with different concentration ratios of (6,5)-chirality CNTs and C.I. Reactive Blue 21, especially [C.I. Reactive Blue 21]/[(6,5)-chirality CNT] = 1, 3, 4.5, 6, 9, and 12 were also conducted. The method of gelation is also the same as described in Section 4.2 for this experiment.

### 4.5. Investigating Electrical Properties of CNT Hydrogels

As described in the Introduction, CNTs have unique electrical properties and are known to behave in a metallic or semiconducting property depending on their structure. In this study, we evaluated whether the electrical properties of CNTs themselves can be preserved when CNT dispersions are gelatinized. Specifically, the conductivity of the CNT hydrogels was measured here using a semiconductor parameter analyzer (Semiconductor Characterization System, 4200A-SCS, Keithley Instruments (Tektronix Inc.), Beaverton, OR, USA). In addition, a simple Metal-Insulator-Semiconductor Field-Effect-Transistor (MISFET) structure was constructed for the hydrogel containing semiconducting CNTs like (6,5)-chirality CNT, and the transistor performance was also measured.

## Figures and Tables

**Table 1:** Results of gelation for each CNT and dispersant combination described in Section 4.1. (✓: gelation, ×: no gelation).

Name	SG65i	HiPco	CG200	CG300	SG101	NC7000
C.I. Reactive Blue 21	✓	×	×	✓	×	×
5,10,15,20-Tetrakis (4-aminophenyl)porphyrin	×	×	×	×	×	×
5,10,15,20-Tetrakis(4-carboxymethyloxyphenyl)porphyrin	×	×	×	×	×	×
Cyanocobalamin (Vitamin B_12_)	✓	×	×	✓	×	×
SDS	×	×	×	×	×	×

**Table 2:** Example results of change in compressive breaking stress when dispersion is heated and cooled.

	Compressive Breaking Stress [kPa]
0 min heating	—
20 min heating	0.3
60 min heating	2.6
180 min cooling after 60 min heating	2.6

**Table 3:** Gelability versus CNT concentration in CNT dispersion. Ratio of CNT to C.I. Reactive Blue 21 mass was fixed at 1:4. (✓: gelation, ×: no gelation).

CNT (wt%)	C.I. Reactive Blue 21 (wt%)	Gelation
0.033	0.13	×
0.067	0.27	×
0.10	0.40	×
0.13	0.53	✓
0.17	0.67	✓
0.27	1.1	✓

**Table 4:** Influence on gelation of changes in ratio of CNT to C.I. Reactive Blue 21. Concentration of CNTs was fixed at 0.1 wt%. (✓: gelation, ×: no gelation).

$\frac{\left[C.I.\;Reactive\;Blue\;21\right]}{\left[\left(6,5\right)-chirality\;CNT\right]}$	Gelation
1	×
3	✓ (at room temperature)
4.5	✓
6	✓
9	✓
12	✓

**Table 5:** Chosen CNTs.

Name	Type	Diameter *	Supplier
SG65i	Single-walled	around 0.78 nm	CHASM, Boston, MA, USA
HiPco	Single-walled	0.8–1.2 nm	NanoIntegris Inc., Boisbriand, QC, Canada
CG200	Single-walled	around 1.3 nm	CHASM, Boston, MA, USA
CG300	Single-walled	around 0.84 nm	CHASM, Boston, MA, USA
SG101	Single-walled	2–3 nm	ZEON CORPORATION, Tokyo, Japan
NC7000	Multi-walled	9.5 nm	Nanocyl SA, Sambreville, Belgium

* From product specification sheets.

**Table 6:** Chosen dispersants.

Name	Structural Formulas	Core Diameter *	Supplier
C.I. Reactive Blue 21	Figure 10a	about 1.5 nm	Santa Cruz Biotechnology, Inc., Dallas, TX, USA
5,10,15,20-Tetrakis (4-aminophenyl)porphyrin	Figure 10b	about 1.9 nm	Tokyo Chemical Industry Co., Ltd., Tokyo, Japan
5,10,15,20-Tetrakis(4-carboxymethyloxyphenyl)porphyrin	Figure 10c	about 2.4 nm	Tokyo Chemical Industry Co., Ltd., Tokyo, Japan
Cyanocobalamin (Vitamin B_12_)	Figure 10d	about 1.5 nm	Sigma-Aldrich/Merck, Darmstadt, Germany
SDS	—	about 1.8 nm (Length)	Nacalai Tesque Inc., Kyoto, Japan

* Estimated by using MolCalc [57].

**Table 7:** Chosen concentration of CNT and C.I. Reactive Blue 21.

CNT (wt%)	C.I. Reactive Blue 21 (wt%)
0.033	0.13
0.067	0.27
0.10	0.40
0.13	0.53
0.17	0.67
0.27	1.1

## Data Availability

The data that support the findings of this study are available from the corresponding author upon reasonable request.
